# Prune belly syndrome in an Egyptian infant with Down syndrome: A case report

**DOI:** 10.1186/1752-1947-2-322

**Published:** 2008-10-02

**Authors:** Kotb A Metwalley, Hekma S Farghalley, Alaa A Abd-Elsayed

**Affiliations:** 1 Department of Paediatrics, Faculty of Medicine, Assiut University, Assiut, Egypt; 2 Department of Paediatrics, Al-Mabarah Hospital, Assiut, Egypt; 3 Department of Public Health and Community Medicine, Faculty of Medicine, Assiut University, Assiut, Egypt

## Abstract

**Introduction:**

Prune belly syndrome is a rare congenital anomaly of uncertain aetiology almost exclusive to males. The association between prune belly syndrome and Down syndrome is very rare.

**Case presentation:**

A 4-month-old Egyptian boy was admitted to our institute for management of acute bronchiolitis. He was born at full term by normal vaginal delivery. His mother, a 42-year-Egyptian villager with six other children, had no antenatal or prenatal care. On examination, the boy was found to be hypotonic. In addition to features of Down syndrome, karyotyping confirmed the diagnosis of trisomy 21. Ultrasound examination of the abdomen showed bilateral gross hydronephrosis with megaureter. Micturating cystourethrography showed grade V vesicoureteric reflux bilaterally with no urethral obstruction. Serum creatinine concentration was 90 μmol/litre, serum sodium was 132 mmol/litre and serum potassium was 5.9 mmol/litre.

**Conclusion:**

We report an Egyptian infant with Down syndrome and prune belly syndrome. The incidence of this association is unknown. Routine antenatal ultrasonography will help in discovering renal anomalies which can be followed postnatally. Postnatal detection of prune belly syndrome necessitates full radiological investigation to detect any renal anomalies. Early diagnosis of this syndrome and determining its optimal treatment are very important in helping to avoid its fatal course.

## Introduction

Prune belly syndrome (PBS) (bilateral gross hydronephrosis, megaureter, and megacystis with abdominal muscle deficiency) is a rare congenital anomaly of uncertain aetiology almost exclusive to males [1,2]. It is caused by urethral obstruction early in development resulting in massive bladder distension and urinary ascites, leading to degeneration of the abdominal wall musculature and failure of testicular descent. The impaired elimination of urine from the bladder leads to oligohydramnios, pulmonary hypoplasia, and Potter's facies. The syndrome has a broad spectrum of affected anatomy with different levels of severity. The exact aetiology of PBS is unknown, although several embryologic theories attempt to explain the anomaly [3]. The association between PBS and Down syndrome (DS) was reported in a few cases. The cause of this association is still unknown. We report a 4-month-old Egyptian boy with PBS and features of DS. Diagnosis was confirmed by karyotyping and micturating cystourethrography.

## Case presentation

**Figure 1:**
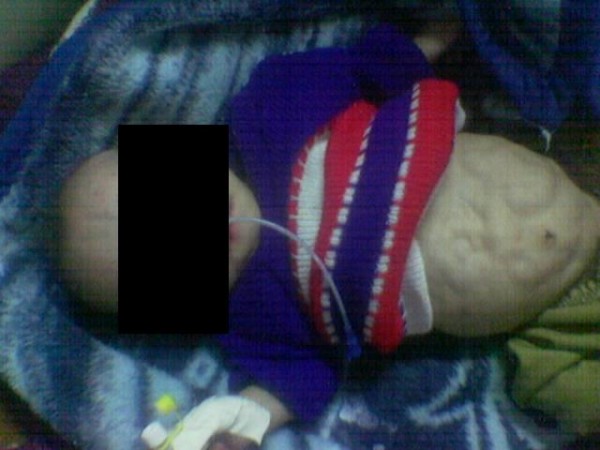
Prune belly syndrome in a child with Down syndrome.

A 4-month-old Egyptian boy was admitted to our pediatric emergency department for management of acute bronchiolitis. He was born at home after full term normal vaginal delivery with no previous hospitalization. His mother, a 42-year-old Egyptian villager with six other children, had no antenatal or prenatal care. On examination, he was found to be hypotonic. In addition to features of DS, karyotyping confirmed the diagnosis of trisomy 21. Abdominal examination revealed a distended abdomen with thin wrinkled skin and visible peristalsis (Figure 1) and with palpable kidneys and bilateral undescended testes. His blood pressure was within the normal range and cardiac examination was normal both by clinical examination and echocardiography. Ultrasound examination of the abdomen showed bilateral gross hydronephrosis with megaureter. Micturating cystourethrography showed grade V vesicoureteric reflux bilaterally with no urethral obstruction. Serum creatinine concentration was 90 μmol/l, serum sodium was 132 mmol/litre and serum potassium was 5.9 mmol/l. The patient died from respiratory failure 5 days after hospital admission.

## Discussion

Renal hypoplasia, hydroureter hydronephrosis, ureterovesical and ureteropelvic junction obstruction, posterior urethral valve and vesicoureteric reflux, have all been associated with DS [4]. PBS has rarely been reported in association with DS [5,6]. Al Harbi reported a similar case of PBS and DS in a girl [7]. Current theories on the pathogenesis of PBS suggest some yet unknown mesodermal injury and or in utero urinary tract obstruction [8]. A genetic cause may also be possible. However, this does not exclude modification of the severity of PBS by the associated chromosomal anomaly [9]. It has been recognized recently that many genes involved in renal nephrogenesis either reappear or are expressed to a markedly greater degree in renal disease [10]. The prognosis of PBS is poor with stillbirths and early infant deaths being common [11]. Diao *et al. *reported that renal failure is the main cause of death in PBS [2]. The lack of prenatal care prevented the analysis of the family pedigree and possible prenatal diagnoses of both syndromes.

## Conclusion

We report an Egyptian infant with DS and PBS. The incidence of this association is unknown, however, there appears to be an incidence of renal and urological anomalies in patients with DS that is higher than previously reported. Routine antenatal ultrasonography will help in discovering renal anomalies which can be followed postnatally. Postnatal detection of PBS necessitates full radiological investigations to detect any renal anomalies. Early diagnosis of this syndrome and determining its optimal treatment are very important in helping to avoid its fatal course.

## Abbreviations

DS: Down syndrome; PBS: Prune belly syndrome.

## Competing interests

The authors declare that they have no competing interests.

## Authors' contributions

KM and AAA-E participated in the clinical management of the case and in manuscript writing. HF participated in the clinical management of the case.

## Consent

Written informed consent was received from the patient's next-of-kin for publication of this case report and any accompanying images. A copy of the written consent is available for review by the Editor-in-Chief of this journal.
